# Education and employment outcomes of young adults with a history of developmental language disorder

**DOI:** 10.1111/1460-6984.12338

**Published:** 2017-11-15

**Authors:** Gina Conti‐Ramsden, Kevin Durkin, Umar Toseeb, Nicola Botting, Andrew Pickles

**Affiliations:** ^1^ School of Health Sciences The University of Manchester and Manchester Academic Health Sciences Centre (MAHSC) Manchester UK; ^2^ School of Psychological Sciences and Health University of Strathclyde Glasgow UK; ^3^ Department of Psychology Manchester Metropolitan University Manchester UK; ^4^ Language and Communication Science City University London UK; ^5^ Department of Biostatistics, Institute of Psychiatry King's College London London UK

**Keywords:** developmental language disorder, education, employment, young adulthood

## Abstract

**Background:**

Developmental language disorder (DLD) presents a considerable barrier for young adults to engage in further education and training. Early studies with young adults with DLD revealed poor educational achievement and lack of opportunities to progress in education. More recent studies have provided more positive findings. Relatively sparse data exist, however, on current cohorts and the factors that predict outcomes.

**Aims:**

To examine educational and employment outcomes in young adulthood in a sample of people with histories of DLD compared with an age‐matched peer group without DLD. We ask: How do educational pathways and early jobs compare between those with and without DLD? Are young adults with DLD receiving similar levels of income as their peers? To what extent are language and literacy abilities associated with outcomes?

**Methods & Procedures:**

Participants included 84 individuals with DLD (67% males) and 88 age‐matched peers without DLD (56% males). Participants were on average 24 years of age. They completed a battery of psycholinguistic, literacy and nonverbal skills assessments. Data were also collected on educational qualifications, current educational status, extent of educational support received, employment status, history and support, as well as current income.

**Outcomes & Results:**

Those with DLD obtained lower academic and vocational qualifications. Higher educational/vocational qualifications were associated with better language, better reading and higher performance IQ (PIQ). There were few differences between the two groups in terms of engagement with education, but the mean age at leaving education was significantly earlier in the participants with DLD. Substantially more participants with DLD reported receiving support or dispensation from their educational institution. There was no significant difference between groups in the proportion of young people currently employed, though a higher proportion of the age‐matched peers was in work full time. Participants with DLD were much more likely to be in non‐professional occupations. However, when examining pay in relation to types of occupation, the groups’ incomes were broadly comparable.

**Conclusions & Implications:**

At the group level, young people with a history of DLD more commonly have less skilled employment and more rarely achieve professional roles. At the individual level there is considerable variation with smaller but not trivial proportions of young adults with a history of DLD showing good educational and employment outcomes. There are positive aspects to early adult outcomes for some young people with a history of DLD.

**What this paper adds**

**What is already known on the subject**

Young people with a history of DLD tend, as a group, to have lower educational outcomes than their peers. They face barriers and disadvantage as they attempt to join the workforce; as a result, smaller proportions obtain full‐time employment.

**What this paper adds to existing knowledge**

This longitudinal study indicates that those with DLD in childhood continue to face difficulties in the later stages of education and the earlier stages of employment, but also that there are signs of ongoing improvements and individual differences. Several positive findings emerge: all participants with a history of DLD achieved some qualifications; many obtained some form of employment; the proportion of individuals who were not in education, employment or training (NEET) was in line with figures for the UK in general; a minority entered professional careers; and early‐career income levels were equivalent to those of peers.

**What are the potential or actual clinical implications of this work?**

Some progress has been made in terms of educational achievement and employment of young people with a history of DLD. The findings of this study underline the need for continued support for these individuals into young adulthood.

## Introduction

Educational and employment outcomes are crucial markers of how adequately a society has prepared its young for adulthood in Western cultures. Of particular interest to those concerned with the development and support of individuals with speech, language and communication difficulties is the question of how these young people's outcomes compare with those of their peers in general. In this study, we examine outcomes with respect to a group of young adults with a history of developmental language disorder (DLD) who attended language units in childhood in the mid to late 1990s, reaching adulthood in the second decade of the 21st century.

Education, training and employment opportunities for young adults have changed considerably in recent decades. In the UK, where the present study was conducted, there has been a steady rise in the proportion of young people engaging in post‐compulsory education or training and/or gaining immediate employment following school, reaching 91% in the latest figures released by the government (Department for Education 2015). National UK statistics reveal the proportion of young people not in education, employment or training (NEET) is at its lowest since records began in 1994.^1^ At the same time, not all educational options and not all employment choices are equal. There is concern that inequalities between different routes to work are incentivising for those who pursue academic options and disadvantageous to those who follow vocational paths (Select Committee on Social Mobility 2016).

### Developmental language disorder (DLD)

Historically, different diagnostic terminology has been used to describe children whose language difficulties are not accounted for by physical, cognitive and/or neurological causes (Bishop 2014, Durkin and Conti‐Ramsden 2010, Reilly *et al*. 2014, 2015). These include language impairment (LI), DLD and specific language impairment (SLI). Longitudinal studies in this area, e.g. the Manchester Language Study (MLS), have also reflected in their publications the historical changes in terminology used with this population (Conti‐Ramsden and Botting 1999). In line with current recommendations, following a Delphi consensus study focusing on characteristics, diagnosis and terminology in this area (Bishop *et al*. 2016a, 2016b), this paper will use the term ‘DLD’ throughout.

DLD is a common developmental disorder (Leonard 2014), characterized by difficulties in the ability to learn and use language (Conti‐Ramsden *et al*. 2012). Tomblin and colleagues’ classic epidemiological study carried out in the United States revealed that DLD affects approximately 7% of children starting school (Tomblin *et al*. 1997, see also Norbury *et al*. 2016 for results with a UK sample).

Although originally thought to be a childhood disorder, research on the trajectory of DLD reveals that it can be persistent, particularly after school entry and in those with both receptive and expressive difficulties (Conti‐Ramsden *et al*. 2009, Howlin *et al*. 2000, Johnson *et al*. 2010, Law *et al*. 2000, Stothard *et al*. 1998). Thus, DLD has immediate consequences but also can have long‐term ramifications in individuals’ lives that go beyond language understanding and use. It is known that individuals with DLD face challenges in a number of areas of functioning through childhood, adolescence and adulthood. Longitudinal studies have indicated that persistent DLD is associated with poorer literacy and academic achievement in adolescence (Durkin *et al*. 2010, Snowling *et al*. 2001), a decline in nonverbal functioning over childhood to adolescence (Conti‐Ramsden *et al*. 2012), and social and psychiatric difficulties in childhood and adolescence (Beitchman *et al*. 2001, Conti‐Ramsden and Botting 2004, Conti‐Ramsden *et al*. 2013, 2016, St Clair *et al*. 2011, Yew and O'Kearney 2013). Despite these known risks, studies on outcomes of individuals with a history of DLD in young adulthood are few in number.

### Education and employment in young adults with DLD: what do we know?

#### Education

Language is central to many areas of educational activity and DLD presents a considerable barrier for young adults with DLD to engage in further education and training. Both reading and writing are areas of difficulty for adolescents with DLD (Bishop and Snowling 2004, Dockrell *et al*. 2007a, St Clair *et al*. 2010). These difficulties are likely to continue into adulthood (Law *et al*. 2009). We know, for example, that adolescents with DLD score lower on standard national tests, and are sometimes less likely to be put forward to take the standard national tests at the end of compulsory education (Conti‐Ramsden *et al*. 2009, Dockrell *et al*. 2007b, Johnson *et al*. 2010, Snowling *et al*. 2001).

Studies based on data collected towards the end of the last millennium revealed poor educational achievement and lack of opportunities to progress in education in young adults with DLD. Rutter and colleagues (Clegg *et al*. 2005, Howlin *et al*. 2000, Mawhood *et al*. 2000) examined the outcomes for a group of 23–24‐year‐old men in the UK who attended schools in the 1970s/80s and found that they all left compulsory education with no educational qualifications and none continued to engage with further education and/or training. Similar negative educational findings were reported in the United States for young adults with DLD who attended school during the same period. These young people left high school and did not progress into any form of further education and/or training (Records *et al*. 1992).

More recent studies, based on data collected in the 2000s, suggest that educational opportunities have improved for young people with disabilities, at least with respect to English‐speaking societies (Department for Education 2016). In the UK, for example, a substantial proportion of young people with DLD now obtain national educational qualifications (Dockrell *et al*. 2007a, Snowling *et al*. 2001, Whitehouse *et al*. 2009). On average, in excess of 40% of adolescents with DLD obtain at least one of the expected qualifications at the end of compulsory education (Conti‐Ramsden *et al*. 2009), and the majority (91%) continue to engage in some form of further education and/or training (Durkin *et al*. 2009). Johnson *et al*. (2010) in Canada also found that 76% of their sample of young adults with DLD completed high school and 27% went on to complete a university undergraduate degree.

#### Employment

In terms of employment, findings for individuals with DLD again highlight a history of barriers and disadvantage. Pathways to work are often initiated during the school years. As young people approach the workforce and are guided into short‐term work placements as part of the latter years of compulsory education, adolescents with DLD are more likely to be placed in lower skilled, elementary jobs (e.g., general supermarket assistant), while their peers without a history of language problems are more likely to be placed in employment that has greater potential for skills development (e.g., classroom assistant; Durkin *et al*. 2010). Some evidence points to poor employment outcomes in early adulthood compared with peers without DLD. For example, the Rutter *et al*. studies (Clegg *et al*. 2005, Howlin *et al*. 2000, Mawhood *et al*. 2000) comparing young adults with autism and young adults with DLD found worse employment outcomes for young adults with DLD despite the fact that the autism group was more disadvantaged (in terms of social and communication skills). Few young adults with DLD were in full‐time jobs and those who were employed tended to have jobs requiring limited education or training, such as street cleaning, security guard, drain maintenance or factory work. Similar negative employment results were found in the United States for a small group of 21‐year‐olds with DLD followed by Tomblin and colleagues (Records *et al*. 1992). More recently in the UK, Whitehouse *et al*. (2009) also found a smaller proportion of adults with a history of DLD aged 16–31 years were in employment when compared with their same‐age peers, and a larger proportion had employment on a part time or temporary basis.

Research based on data collected in the 2000s suggest that relatively less skilled, lower status positions in the manual, service and retail sectors are more common for young adults with DLD than for their peers in the UK (Carroll and Dockrell 2010, 2012, Conti‐Ramsden and Durkin 2012, Roulstone and McLeod 2011). Similarly in Canada, Johnson *et al*. (2010) found that 25‐year‐old individuals with DLD held jobs that were classifiable as lower in socio‐economic status (SES) than the jobs held by their peers. These investigators report, for example, that while young people with DLD, as well as their peers, found jobs within the food service industry, the ratio of those employed as servers (e.g., waitress, bartender) to those employed as managers (e.g., restaurant managers) was 9:1 in the DLD group, while in the comparison group without DLD the ratio was 6:4. In Denmark, a 30‐year follow‐up study of young people originally diagnosed with DLD in childhood also revealed unemployment at rates higher than in the general population (Elbro *et al*. 2011).

### The present study

Educational and employment prospects, then, can be circumscribed by DLD. It is important to keep in mind that disadvantageous group comparisons with peers without DLD hold *on average*. There are exceptions, and some young adults with DLD do progress well in education, training and employment (Dockrell *et al*. 2007b, Durkin *et al*. 2009, Johnson *et al*. 2010). Two types of factors may be particularly influential. One may be the severity of the young adults’ difficulties, including language, reading and nonverbal skills; the other is the support received both professionally, from their managers or colleagues in employment, and personally, from family and friends (Carroll and Dockrell 2012, Clegg *et al*. 2012, Conti‐Ramsden *et al*. 2009, Dockrell *et al*. 2007, Durkin *et al*. 2009, Snowling and Hulme 2012). We acknowledge there are other factors that are likely to be associated with poor language skills and education/employment outcomes, notably social factors (Bradshaw 2016). In this investigation, we examine both individual profiles of abilities and education/employment support received as predictors of educational and employment outcomes in 24‐year‐old adults with DLD. We also designed the study taking into consideration potential contributing variables (e.g., social factors) so that comparisons across individuals with DLD and their peers were warranted.

This investigation makes two distinctive further contributions. First, we examine GCSE grades as predictors of educational and employment outcomes. GCSEs are nationally regulated, internationally recognized qualifications in specific subjects (e.g., English, Maths, Biology, Chemistry, French). Children in the UK study for GCSEs when they are aged 14–16 years. GCSE grades are predictive of future educational performance (Huws *et al*. 2006, McDonald *et al*. 2001). In the government's National Qualifications Framework (table 2) a distinction is drawn between Level 1 attainments (GCSEs at grades D–G) and Level 2 attainments (GCSEs at the higher grades C–A*). At the time of this study, obtaining five or more GCSEs at the higher grades was a ‘gateway’ attainment that enable the young person to proceed to AS and A Level qualifications, taken at around ages 17–18, classed as Level 3 in the National Framework and, in turn, the basis for selection for higher educational courses (Levels 5 and 6). Approximately 16% of the young people with DLD in the present sample had reached Level 3 at ages 16–17 years, which is consistent with national statistics on the proportion of children with special educational needs reaching this level (14.1%; Conti‐Ramsden *et al*. 2009).^2^

Given the changes in educational opportunities and the findings that young adults with DLD are nowadays more likely to obtain GCSE qualifications than before (Conti‐Ramsden *et al*. 2009), this investigation undertakes what we believe to be the first examination of GCSE grades as predictors of educational outcomes in young adults with DLD; it extends this approach to examine also whether GCSE grades are predictive of employment outcomes in these individuals.

The second distinctive contribution of this study is that we examine income earned in employment. There is relatively little previous evidence on income in people with DLD. Records *et al*. (1992) did compare income levels in 29 young American adults (17–25‐year‐olds, mean age 21 years) with DLD and 29 non‐affected peers, and found no significant differences. However, this was a small sample, with the majority still attending school or college and only about 25% in employment, meaning that income levels were predominantly low. Furthermore, of those who were employed, most of the DLD group were in full‐time employment while most of their comparison peers were in part‐time employment (presumably consistent with ongoing study commitments); thus, the comparability of group income levels needs to be interpreted in light of the fact that one group (DLD) was working full time to match the income of a group working part time. Additional data are clearly needed. The situation is also influenced by the fact that, in the UK, the Equality Act came into force in 2010, specifying guidance for employers including equal pay for individuals with disability.^3^ To our knowledge, the present study is the first to compare income in young adulthood for young people with and without DLD in the light of this legislation.

The purposes of this investigation, then, were to examine educational and employment outcomes in young adulthood in the 2010s for a sample of people with histories of DLD. The data for this study were collected between 2012 and 2015. We compare participants with DLD with an age‐matched peer group without DLD, controlling for a number of variables to afford meaningful comparisons across groups. We ask: How do the educational pathways and early jobs of those with DLD compare with those of peers without DLD? Are young adults with a history of DLD receiving similar levels of income as their peers? To what extent are language and literacy abilities associated with outcomes?

## Method

### Ethics

The study reported here received ethical approval from the University of Manchester. All participants provided informed written consent.

### Participants

#### Participants with a history of DLD

Participants were young adults with a history of DLD who were originally part of a wider study (Conti‐Ramsden and Botting 1999, Conti‐Ramsden *et al*. 1997): the Manchester Language Study (MLS). The initial cohort of 242 children (23% female) were recruited from 118 language units across England and represented a random sample of 50% of all 7‐year‐olds attending language units for at least half the school week. Language units are specialized classes for children who have been identified with DLD, i.e., primary language difficulties. Language unit placements were offered to children who would find it difficult to cope in mainstream education even with support. These children are deemed to need a structured small group setting with intensive language input that usually involves both teachers and speech and language therapists. Children with frank neurological difficulties, hearing impairment, a diagnosis of autism or a general learning disability were excluded.

Individuals were contacted and reassessed again at ages 8 (*N* = 232), 11 (*N* = 200), 14 (*N* = 113), 16 (*N* = 139), and 24 (*N* = 84).

Sample attrition is a common problem in longitudinal studies, and the MLS is no exception. The attrition observed in the MLS sample was partly due to funding constraints at follow‐up stages of the study, which arguably is less likely to introduce bias. Nonetheless, we know that some language and cognitive change occurs in DLD from childhood to young adulthood (Conti‐Ramsden *et al*. 2012, Botting 2005) which may lead to a selective (or biased) sample of individuals with particular profiles continuing to participate (e.g., those with most persistent language difficulties). We therefore examined the psycholinguistic characteristics of the 84 participants at various earlier points in their development and compared them with individuals who did not participate in the MLS in adulthood.

The current sample, 35% of the original cohort, consisted of 56 (67%) men and 28 (33%) women ranging in age from 23.4 to 25.9 years (mean = 24.4; standard deviation (SD) = 0.65 years). There were no significant differences in receptive language (*t*(240) = −1.13, *p* = .261), expressive language (*t*(229) = −0.45, *p* = .654), and nonverbal IQ (*t*(231) = −0.60, *p* = .547) standard scores at age 7 between those who participated at age 24 and those who did not. Similarly, there were no significant differences in receptive language (*t*(240) = −0.87, *p* = .389), expressive language (*t*(229) = −0.64, *p* = .521), and nonverbal IQ (*t*(231) = −0.19, *p* = .851) standard scores at age 7 between those who participated at age 16 and those who did not. We also compared educational data at age 16 between those who participated at age 24 and those who did not. As can be seen from table 1, there were no significant differences in GCSE points, number of entry‐level qualifications, number of Level 1 or 2 qualifications nor the proportion of core GCSEs taken between those who did/did not participate at age 24. Given all the above comparisons, there is evidence to assume that the DLD sample participating in this study at 24 years is representative of the MLS cohort as a whole.

**Table 1:** Comparison of key educational variables at age 16 years for participants with developmental language disorder (DLD) who did/did not participate at age 24 years

	Participated at 16 but not 24, mean (SD)/*n* (%)	Participated at 16 and 24, mean (SD)/*n* (%)	Test statistic
GCSE points (0–676)	166.66 (153.79)	195.63 (145.00)	*t*(130) = −1.09^NS^
Number of entry‐level qualifications (0–13)	1.44 (2.29)	1.49 (2.82)	*t*(130) = −0.10^NS^
Number of Level 1 qualifications (0–11)	3.67 (3.51)	4.31 (3.06)	*t*(130) = −1.11^NS^
Number of Level 2 qualifications (0–14)	1.53 (2.77)	1.82 (2.72)	*t*(132) = −0.59^NS^
Highest academic qualification (Levels 1–3)	2.13 (0.82)	2.33 (0.74)	*t*(128) = −1.46^NS^
Core GCSE's taken? (English, Maths, and Science), yes/no	35 (67%)	66 (81%)	*χ* ^2^ (1, *N* = 133) = 3.48^NS^

Note: NS, not significant. There were 16 participants with DLD who had vocational qualifications (Levels 1–3). Those who participated at 16 and 24 obtained on average a lower level vocational qualification (mean = 1.9, SD = 0.3) than those who only participated at age 16 years (mean = 2.4; SD = 0.5), *t*(14) = 2.48. *p* < .05.

#### Age‐matched peers (AMP)

In order to make meaningful comparisons between participants with DLD and their AMP, it is important to demonstrate that the characteristics of the AMP are closely matched to that of the participants with DLD (with the exception of their language profiles). To this end we put in place a targeted participant strategy in order to achieve comparability on factors such as gender, social data and wherever possible, type of school (see below). The comparison group thus consisted of 88 AMP who had no history of special educational needs or speech and language therapy provision. Forty‐nine (56%) were men and 39 (44%) were women, ranging in age between 22.3 and 25.9 years (mean = 24.1 years; SD = 0.90 years). The gender distribution of the DLD group (67% male; 33% female, described above) was not significantly different from the gender distribution of the AMP group, *χ* ^2^(1, *N* = 172) = 2.2, *p* = .140. Sixty‐six of these young adults were recruited from the MLS age‐matched comparison cohort first established when the individuals were 16 years of age. Age‐matched participants without a history of DLD recruited at age 16 came from the same schools as the participants with a history of DLD as well as additional targeted schools. These participants were sampled from selected demographic areas in order to ensure AMPs came from broad background and wide geographical areas, similar to participants with a history of DLD. Twenty‐two young adults were recruited for the current investigation. The additional 22 young adults were recruited to ensure comparability with the DLD sample participating in this study in terms of age and SES as measured by personal income. The DLD and the AMP groups did not differ on household income at age 16 years (*χ* ^2^(10, *N* = 145) = 9.32, *p* = .501) nor personal income at age 24 years (*χ* ^2^(5, *N* = 131) = 7.38, *p* = .194). In addition, comparisons were carried out between the DLD and AMP groups on additional key social factors at age 16 including maternal education, other languages spoken at home and whether parent was a homeowner. We found no significant differences across those with and without DLD (table 2). As expected, language, performance IQ (PIQ) and reading profiles were different across the two groups (table 3).

**Table 2:** Comparison of DLD and age‐matched peers (AMP) groups on social factors at age 16 years

	DLD, *n* (%)	AMP, *n* (%)	Test statistic
Mother achieved at least one GCSE qualification?	62 (47%)	57 (48%)	*χ* ^2^ (1, *N* = 251) = 0.07^NS^
Mother achieved at least one A‐Level qualification?	56 (42%)	54 (46%)	*χ* ^2^ (1, *N* = 251) = 0.34^NS^
Mother achieved at least a university qualification?	19 (14%)	20 (17%)	*χ* ^2^ (1, *N* = 251) = 0.34^NS^
Other languages spoken at home?	10 (7%)	6 (5%)	*χ* ^2^ (1, *N* = 254) = 0.55^NS^
Parent homeowner?	99 (75%)	95 (82%)	*χ* ^2^ (1, *N* = 248) = 1.72^NS^

Note: Values represent the number of participants answering yes with per cent in parentheses.

**Table 3:** Psycholinguistic profiles for the two groups of participants at age 24 years

	DLD		AMP				
	Mean (SD)	Range	Mean (SD)	Range	*t*	d.f.^a^	Mean difference [95% CI]
Receptive language (SS)	83.5 (18.6)	55–115	106.2 (8.9)	65–115	−10.2***	168	−22.7 [−27.1, −18.3]
Receptive language (RS)	15.82 (5.06)	1–24	21.72 (2.23)	12–24	−9.88***	168	−5.90 [−7.08, −4.72]
Expressive language (SS)	81.6 (18.9)	55–120	105.6 (12.1)	55–120	−9.90***	167	−24.1 [−28.8, −19.3]
Expressive language (RS)	43.41 (9.35)	4–56	51.94 (5.45)	10–56	−7.28***	167	−8.53 [−10.85, −6.22]
Core language (SS)	69.3 (20.7)	40–115	100.0 (13.9)	56–124	11.2***	167	30.6 [25.3, 36.0]
Core language (RS)	115.28 (30.88)	33–174	159.19 (21.98)	34–189	–10.67***	167	−43.90 [−52.02, −35.79]
Nonverbal IQ (SS)	98.8 (15.8)	55–131	111.9 (10.3)	79–129	6.4***	167	13.1 [9.1, 17.2]
TOWRE reading (SS)	79.6 (9.8)	57–111	90.9 (10.7)	66–113	6.8***	148	11.4 [8.1, 14.7]
WORD accuracy (RS)	43.7 (7.6)	19–55	52.2 (3.3)	34–55	9.5***	168	8.5 [6.7, 10.3]
WORD comprehension (RS)	25.4 (6.0)	8–38	31.9 (3.1)	23–38	6.5***	167	6.5 [5.0, 7.9]
Reading overall (RS)	34.5 (6.1)	14–45	42.1 (2.8)	31–47	10.5***	167	7.6 [6.1, 9.0]

Notes: ^***^ *p* < .001. DLD, developmental language disorder; AMP, age‐matched peers; SS, standard score; RS, raw score.

a For all measures with d.f. of 168 there were *n* = 169 participants, d.f. 167 there were *n* = 168 participants, and d.f. 148 there were *n* = 149 participants. The range of *n* within each group across measures was similar, DLD *n* = 79–84 participants and AMP *n* = 71–86 participants.

#### Psycholinguistic assessments of language, reading and nonverbal skills

The Clinical Evaluation of Language Fundamentals (CELF‐4^uk^; Semel *et al*. 2006) was used to assess language ability. Standard scores were calculated using the Word Classes receptive subscale for receptive language and the Formulated Sentences subscale for expressive language. Given the dearth of standardized language tests in adulthood, the CELF‐4 was deemed the best fit assessment for our cohort at 24 years of age (neither group reached ceiling levels on this assessment which is normed up to age 21;11 years). For the age range 17;0–21;11 years, the reliability of the word classes subtest was .88 and of the formulated sentences subtest was .82. Clinical validation studies of the CELF‐4 reported in the manual indicate that the test is sensitive to LI in children, adolescents and young adults. A core language index was calculated using the relevant subscales according to the CELF manual.

The Wechsler Abbreviated Scale of Intelligence (WASI; Wechsler 1999) Performance subscale was administered as a measure of nonverbal IQ and standard scores were calculated. This test has norms for individuals aged 6–89 years. The reliability of the PIQ scale for the age range 20–24 years is .94. Validity studies of the WASI reported in the manual provide evidence that the test is a valid quick screening measure of intellectual functioning.

The Test of Word Reading Efficiency (TOWRE; Torgesen *et al*. 1999) was administered as an overall measure of reading ability that afforded calculating standard scores. The TOWRE has been normed from age 6 to 24;11 years. Standard scores were calculated using the sight word efficiency subtest. The reliability of this subtest for the older age group was .82 (form A) and .87 (form B). Validity studies of the TOWRE reported in the manual provide evidence that the TOWRE is a valid measure of reading, especially when assessing individuals for whom rate of reading is a potential problem.

In addition, we were interested in obtaining more detailed profiles of individuals’ reading abilities, in particular with regard to reading comprehension. For this purpose we used the Basic Reading and the Reading Comprehension subtests of the Wechsler Objective Reading Dimensions (WORD; Wechsler 1993) to measure reading accuracy and reading comprehension, respectively. However, this test only provides normative data up to 16;11 years, thus we used raw scores for analyses at age 24 and these are reported in table 3. An overall score, named Reading Overall, was included as the mean of the two WORD subscales.

The mean standard scores, SDs and DLD versus AMP comparisons on the language, reading and nonverbal measures are presented in table 3. The AMP participants had mean receptive, expressive and reading scores within the expected range. The participants with a history of DLD had significantly lower receptive and expressive language; mean scores fell below 1 SD below the mean (< 85). Interestingly, the range of language scores for both groups of young adults was wide, i.e., there were high language scores for some participants in the group with a history of DLD as well as low language scores of some participants in the AMP group. Both groups had mean nonverbal skills within the expected range. It should be noted, nonetheless, that the young adults with a history of DLD had significantly lower nonverbal IQ scores than their peers, as is often found in research with this population (Leonard 2014). The mean reading scores for young adults with a history of DLD was significantly lower than the mean reading scores for their peers. However, the reading scores indicated that the group with a history of DLD had an average reading age of 11–12 years which was judged to be adequate for understanding the interview questions and statements used in this study. In addition, we took additional steps to facilitate comprehension (see the procedure below).

### Materials used to examine education and employment

#### Education

##### Highest qualifications

Participants were asked about the academic and vocational qualifications they had obtained. The interview was structured following the levels from the National Qualifications Framework (NFQ). The NQF is a system of standardizing qualifications and grouping them according to difficulty. The framework ranges from ‘Entry Level’, which is the lowest level of qualification, to ‘Level 8’, which is the highest (table 4 has full details of the levels and their corresponding qualifications). The key milestone levels are Level 3, A‐Levels and Level 6 and Bachelor's degree. It is also of interest to note Level 1 qualifications, the first level at which individuals are able to apply learning without guidance or supervision. Since data were collected for the present study, the NQF has been substituted in the UK with a very similar system called the Qualifications and Credit Framework.^4^ The highest level of academic qualifications and vocational qualifications obtained were recorded.

**Table 4:** National qualifications framework and equivalent levels

Level	Examples of qualifications	Description
Entry level	Entry‐level certificate Entry‐level Skills for Life	Entry‐level qualifications recognize basic knowledge and skills and the ability to apply learning in everyday situations under direct guidance or supervision. Learning at this level involves building basic knowledge and skills and is not geared toward specific occupations
Level 1	GCSE (grades D–G) NVQ Level 1	Level 1 qualifications recognize basic knowledge and skills and the ability to apply learning without guidance or supervision. Learning at this level is about activities that mostly relate to everyday situations and may be linked to job competence
Level 2	GCSE (grades A^*^–C) NVQ Level 2	Level 2 qualifications recognize the ability to gain a good knowledge and understanding of a subject area of work or study, and to perform varied tasks with some guidance or supervision. Learning at this level involves building knowledge and/or skills in relation to an area of work or a subject area and is appropriate for many job roles
Level 3	AS and A Levels NVQ Level 3	Level 3 qualifications recognize the ability to gain and, where relevant, apply a range of knowledge, skills and understanding. Learning at this level involves obtaining detailed knowledge and skills. It is appropriate for people wishing to go to university, those working independently, or, in some areas, people supervising and training others in their field of work
Level 4	Certificate of Higher Education NVQ Level 4	Level 4 qualifications recognize specialist learning and involve detailed analysis of a high level of information and knowledge in an area of work or study. Learning at this level is appropriate for people working in technical and professional jobs, and/or managing and developing others
Level 5	BTEC Professional Award, Certificate and Diploma Level 5 Diploma of Higher Education	Level 5 qualifications recognize the ability to increase the depth of knowledge and understanding of an area of work or study to enable the formulation of solutions and responses to complex problems and situations. Learning at this level involves the demonstration of high levels of knowledge, a high level of work expertise in job roles, and competence in managing and training others. Qualifications at this level are appropriate for people working as higher‐grade technicians, professionals or managers
Level 6	BTEC Advanced. Professional Award, Certificate and Diploma Level 6 Bachelor's degree	Level 6 qualifications recognize a specialist high‐level knowledge of an area of work or study to enable the use of an individual's own ideas and research in response to complex problems and situations. Learning at this level involves the achievement of a high level of professional knowledge and is appropriate for people working as knowledge‐based professionals or in professional management positions
Level 7	BTEC Advanced. Professional Award, Certificate and Diploma Level 7 Master's degree	Level 7 qualifications recognize highly developed, advanced and complex levels of knowledge, which enable the development of in‐depth and original responses to complicated and unpredictable problems and situations. Learning at this level involves the demonstration of high‐level specialist professional knowledge and is appropriate for senior professionals and senior managers
Level 8	NVQ Level 5 Doctorate	Level 8 qualifications are awarded for the creation and interpretation, construction, and/or exposition of knowledge which extends the forefront of a discipline, usually through original research

Note: GCSE, General Certificate of Secondary Education; BTEC, Business and Technology Education Council; NVQ, National Vocational Qualification; A‐Levels, Advanced Levels; HNC, Higher National Certificate.

##### Education status

Participants were asked whether they were currently in education and if so, whether this was full time. For those who were not currently in full‐time education, we asked whether they had been in education in the last 6 months, and the age at which they left. All participants were asked if they had ever dropped out of a course.

##### GCSE results

To calculate a score which represented both the quality and quantity of academic attainments at the end of compulsory education, grades were converted into numeric scores using the point system used in the education system in the UK at the time the exams were taken. For example, in the aforementioned system in the UK a GCSE at grade A is given 52 points whilst a GCSE at C grade is given 40 points. Thus, a student with two GCSEs at grade C and one at grade A would have a total score of 132 (40 + 40 + 52). Higher scores indicate higher achievement (see also Conti‐Ramsden *et al*. 2009).

It is important to acknowledge that the GCSE grades were based on individual reports and were not verified independently. However, individuals on interview often looked for their certificates and used these as a basis of reporting during the interview. These were observations which were not recorded systematically; thus it is not possible to provide an accurate figure of the proportion of reports which were based on having certificates ‘at hand’.

##### Education support

With reference to their most recent qualification, participants were asked whether they had received institutional support (e.g., extra time for educational exams), whether they had received non‐institutional support (i.e., family or friends), the types and sources of support (participants were asked to select from a list of options, all that apply), and whether they felt that they received the right type of support. For the analyses total support was calculated in a scale of 0–2, where 0 is no support and 2 is both institutional and non‐institutional support received. Note that there was no independent verification of the actual support received by individuals. This is a limitation of the study that calls for future research that includes independent validation as well as information regarding expectations of support in the population as a whole.

#### Employment

##### Employment status

Participants were asked about their current employment situation and responded by choosing one of the following options: not in education, employment, or training (NEET), in full‐time employment, in part‐time employment or other (e.g., in full‐time or part‐time education, self‐employed).

##### Employment history

Participants were asked how many paid jobs they had held since leaving full‐time education. For those participants who were currently unemployed, the duration of unemployment was established. Participants were also asked to rate on a scale of 1–5 (where 1 is very unlikely and 5 is very likely), the likelihood of obtaining employment in the next 12 months. For those who were currently employed, participants were asked to indicate how many jobs they currently held, duration of their current employment and their job title (which was coded and classified as professional or not based on the Standard Occupational Classification System (SOC); Office of National Statistics (ONS), 2000). The SOC was used to classify occupations into groups and was reverse scored for ease of understanding, Thus, the scale ranged from 1 (elementary, less skills based occupations) to 9 (highly qualified occupations, e.g., solicitors, managers, directors, senior officials). Participants were also asked the number of hours they worked per week in their main job, whether the job was permanent, and whether they were part of a private/company pension. Note that at the time of the interviews, pension contributions were not compulsory for all employees (this was introduced by the British government in 2016). For those in permanent employment, participants were asked to indicate whether they thought they would still be in employment in 12 months.

##### Experiences of gaining employment

Participants were asked to indicate whether they had used a CV to apply for jobs, and whether they had attended an assessment centre, a face‐to‐face interview and/or a telephone interview as part of the application process. In the UK, an assessment centre is a series of structured, timed exercises designed to simulate the activities the applicant is likely to be doing in the job itself.^5^

Participants were asked to rate on scale of 1–5 (where 1 is very difficult and 5 is very easy) the difficulty of the assessment/ interview process. The number of participants who had used the guaranteed interview scheme and/or asked for special arrangements at interview was also recorded.

##### Employment support

For those currently in employment, participants were asked whether they had received support from managers or colleagues, whether this was formal support, and whether they currently had any problems with colleagues or line managers. For the analyses, total support was calculated using a binary variable, 0 for no support and 1 for support received.

##### Income

Participants were asked about their income from all sources of employment and their response was coded into one of the following income bands: < £5200, £5201–£10,400, £10,401–£15,600, £15,601–£20,800, £20,801–£26,000, £26,001–£31,200 or > £31,201. The same income bands were presented as their weekly equivalent amounts so that participants paid on this basis could easily identify their income bracket.

### Procedure

The participants were interviewed face to face at their home on the above measures as part of a wider battery. Interviews took place in a quiet room, wherever possible with only the participant and a trained researcher present. Basic demographic information was collected and then the standardized assessments were administered in the manner specified by the test manuals. For the interview, the items were read aloud to the participants and the participants were given additional clarification, where needed, although this occurred rarely. Particular care was taken to ensure the participants understood the interview items. The response options were carefully explained and both the items and response options were also presented visually. Participants could respond verbally or by pointing to the response options.

### Statistical analysis

All statistical analyses were conducted in Stata/SE 13.1 (StataCorp 2013) and a two‐tailed significance level of *p* = .05 was used unless otherwise specified. Independent *t*‐tests and Chi‐squared (*χ* ^2^) tests were used to compare group differences (or Fischer's exact test for smaller expected cell sizes). Missing data were treated as such and only the available data were analyzed. When reporting percentages, we used a rounding up procedure in order to make the information more accessible and reader friendly (e.g., 55.5% is reported as 56%) thus some of the percentages presented in the tables may add up to 101% as opposed to 100%.

Ordered logistic regression analysis was carried out to investigate the effect of occupational category and DLD status on income. Income was entered as the ordinal outcome variable and the predictors were main effects of occupational category and DLD status. The interaction between employment type and DLD status was not tested due to small numbers of participants in some occupational categories. Following this, to examine whether young people with a history of DLD received similar income levels with comparable education levels and types of employment, two further ordered logistic regression analyses were carried out (income as the outcome variable for both). For the first regression model, predictors were main effects of group and employment type (full time, part time and other). For the second regression model main effects were group and highest educational qualification. Due to small numbers of participants in some of the categories, interactions were not tested.

Zero‐order correlations were run to examine concurrent relationships between the variables of interest. Finally, linear regression models were run to establish whether there was a relationship between GCSE performance and PIQ (independent variables) and education and employment outcomes (dependent variables) at age 24.

## Results

### Highest qualifications

Table 5 shows the highest level of qualification achieved by participants by the age of 24. Fisher's exact tests showed that there were significant differences between the groups for academic qualifications (*p* < .001) and vocational qualifications (*p* < .001).

**Table 5:** Highest academic and vocational qualification

	Highest academic qualification		Highest vocational qualification	
	DLD, *n* (%)	AMP, *n* (%)	DLD, *n* (%)	AMP, *n* (%)
Level 8	0 (0%)	0 (0%)	0 (0%)	0 (0%)
Level 7	1 (1%)	7 (8%)	0 (0%)	1 (1%)
Level 6	7 (8%)	29 (33%)	0 (0%)	2 (3%)
Level 5	1 (1%)	2 (2%)	1 (1%)	4 (5%)
Level 4	1 (1%)	1 (1%)	0 (0%)	2 (3%)
Level 3	5 (6%)	24 (27%)	22 (26%)	23 (26%)
Level 2	32 (38%)	25 (28%)	28 (33%)	16 (18%)
Level 1	22 (26%)	0 (0%)	13 (15%)	1 (1%)
Entry	5 (6%)	0 (0%)	7 (8%)	0 (0%)
None	10 (12%)	0 (0%)	13 (15%)	39 (44%)

#### Academic

The number of young people with DLD achieving at least an academic Level 6 (10%), which is equivalent to an undergraduate degree, was significantly lower than the number of AMPs obtaining this level of academic qualifications (41%), *χ* ^2^ (1, *N* = 172) = 22.2, *p* < .001. In the same vein, the number of young people with a history of DLD achieving at least an academic Level 3 qualification (18%), which is the equivalent of A‐Levels, was significantly lower than their AMP (72%), *χ* ^2^ (1, *N* = 172) = 50.1, *p* < .001. In terms of Level 1, only young people with DLD (26%) reported achieving Level 1 as their highest academic qualification (all AMPs achieved academic qualifications higher than Level 1).

#### Vocational

In contrast to the differential concerning Level 3 academic qualifications, the groups did not differ significantly in the proportions of individuals who obtained Level 3 vocational qualifications (DLD 27% versus AMP 36%), *χ* ^2^(1, *N* = 172) = 1.6, *p* = .207.

However, the spread of vocational qualifications was different from that found for academic qualifications. No individuals with DLD obtained vocational qualifications at Levels 6, 7 or 8, and only a very small proportion of AMPs had proceeded to these levels. We thus examined the proportion of individuals with vocational qualifications at Level 4 or above. There were significantly fewer individuals with a history of DLD who obtained vocational qualifications at or above Level 4 (DLD 1% versus AMP 10%, *χ* ^2^(1, *N* = 172) = 6.4, *p* = .011). In terms of Level 1, the pattern observed was similar to that of academic qualifications (DLD = 16%; AMP = 1%). All participants had some qualifications. There were no individuals in either group who had no qualifications whatsoever.

### Education status

As shown in table 6, there were few differences between the two groups in terms of engagement with education, with a couple of exceptions. The proportion of participants with DLD who were in any education (full time or part time) in the preceding 6 months was significantly lower than the corresponding proportion of the AMP group. The proportion of participants with DLD who reported ever having dropped out of education was slightly lower than the proportion of AMPs who had done so, though this was a difference of borderline significance (*p* = .06). For those who were not currently in full‐time education, the DLD group (mean = 18.7, SD = 2.1) left education at a significantly earlier age than their AMPs (mean = 19.7, SD = 2.5), *t*(154) = −2.6, mean difference = −1.0 [95% CI = −1.7, −0.2], *p* < .05, Cohen's *d* = −0.4.

**Table 6:** Education status and support

	DLD, *n* (%)	AMP, *n* (%)	*χ* ^2^ statistic
*Education status*			
Currently in education?	15 (18%)	27 (31%)	*χ* ^2^(1, *N* = 171) = 3.7^NS^
Full time?	5 (33%)	15 (56%)	*χ* ^2^(1, *N* = 42) = 1.9^NS^
In education last 6 months?	16 (19%)	29 (33%)	*χ* ^2^(1, *N* = 170) = 4.3*
Ever dropped out?	16 (19%)	28 (32%)	*χ* ^2^(1, *N* = 171) = 3.5^NS^
*Educational support*			
Institutional support?	36 (44%)	7 (8%)	*χ* ^2^(1, *N* = 169) = 29.6***
Other support?	31 (39%)	25 (28%)	*χ* ^2^(1, *N* = 168) = 2.0^NS^
Enough support?	52 (83%)	25 (81%)	*χ* ^2^(1, *N* = 94) = 0.1^NS^
Right type of support?	52 (83%)	29 (97%)	*χ* ^2^(1, *N* = 93) = 3.6^NS^

Note: NS (not significant) and % represent those who answered yes. ^*^ *p* < .05, ^**^ *p* < .01, ^***^ *p* < .001.

### Educational support

As would be expected, substantially more participants with DLD reported receiving institutional support (table 6). The type of institutional support received was extra time in exams (DLD = 19, AMP = 5), help with writing (DLD = 14, AMP = 1), help with reading (DLD = 17, AMP = 0), help with computer use (DLD = 2, AMP = 2), and other unspecified support (DLD = 16, AMP = 4).

In terms of non‐institutional support, there was no significant difference between the groups. Sources of non‐institutional and informal support were partner (DLD = 4, AMP = 4), parent (DLD = 16, AMP = 12), sibling (DLD = 5, AMP = 1), other relative (DLD = 2, AMP = 1), friends (DLD = 5, AMP = 4), other (DLD = 3, AMP = 4), other students/tutors (DLD = 7, AMP = 4), and colleagues (DLD = 2, AMP = 2). Participants sought support with proof reading (DLD = 10, AMP = 13), writing (DLD = 14, AMP = 3), reading (DLD = 8, AMP = 0), computer use (DLD = 11, AMP = 0), and other unspecified support (DLD = 19, AMP = 16). There was no difference in the percentage of individuals in each group that felt they had received enough support and that they had received the right type of support.

### Employment status

**Figure 1:**
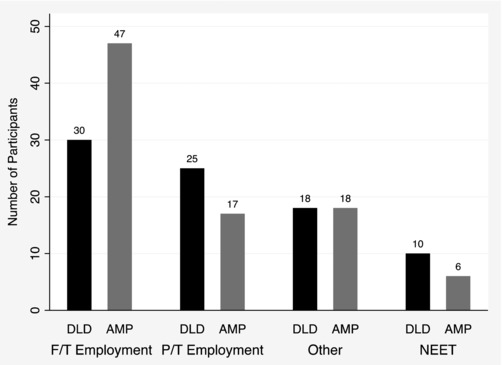
Employment status.

Figure 1 shows the breakdown of employment status for each of the groups (NEET: DLD = 12% versus AMP = 7%, full‐time employment: DLD = 36%, versus AMP = 53%, part‐time employment DLD = 30% versus AMP = 19%, other DLD = 22% versus AMP = 20%). There was no significant difference for the % of young people in each of the groups currently in employment (DLD = 66%, AMP = 73%, *χ* ^2^(1, *N* = 171) = 0.8, *p* = .359). There were no significant differences between the group in terms of the number of NEETs, *χ* ^2^(1, *N* = 171) = 1.4, *p* = .241, nor those in part‐time employment, *χ* ^2^(1, *N* = 171) = 2.7, *p* = .101, nor those falling in the ‘other’ category of employment, *χ* ^2^(1, *N* = 171) = 0.0, *p* = .843. There were, however, significantly more AMPs in full‐time employment compared with the DLD = group, *χ* ^2^(1, *N* = 171) = 5.1, *p* = .023.

Table 7 presents the distributions of each group across occupational categories, as defined by the Standard Occupational Classification System (SOCS). There was a significant difference between the groups in the types of occupations (Fisher's exact *p* = .013). The data in table 5 indicate an imbalance such that there are few participants (10%) with DLD in the professional occupations (categories 7–9) whilst almost 40% of the AMPs have these kinds of employment positions. In the same vein, 90% of the DLD group fell into the non‐managerial, less skilled occupational categories (categories 1–6). A chi square test confirmed that this difference was significant, *χ* ^2^(1, *N* = 131) = 14.7, *p* < .001.

**Table 7:** Categories of employment by group

	DLD, *n* (%)	AMP, *n* (%)
1. Elementary occupations	8 (14%)	5 (7%)
2. Process, plant, and machine operatives	2 (3%)	1 (1%)
3. Sales and customer service occupations	19 (32%)	12 (17%)
4. Caring, leisure, and other trades occupations	9 (15%)	8 (11%)
5. Skilled traders occupations	7 (12%)	4 (6%)
6. Administrative and secretarial occupations	9 (15%)	13 (18%)
7. Associate professional and technical occupations	3 (5%)	9 (13%)
8. Professional occupations	2 (3%)	15 (21%)
9. Managers, directors, senior officials	1 (2%)	4 (6%)

### Employment history

Table 8 compares the jobs histories of the participants by group. There were no significant differences between the groups for the number of paid jobs held since leaving full‐time education. For those who were unemployed, young people with a history of DLD had been unemployed for significantly longer and rated themselves as significantly less likely to gain employment in the next 12 months. In addition, we found that for those who were employed, there was no significant difference between the groups for the number of jobs they currently held, the duration of current employment, the number of hours worked per week, whether they were part of a private/company pension scheme, nor whether they were in permanent employment. For those in permanent employment there was no significant difference in whether they expected still to be permanently employed in 12 months,

**Table 8:** Employment history

	DLD, mean (SD)/*n* (%)	AMP, mean (SD)/*n* (%)	Mean difference [95% CI]	Test statistic	Effect size
Number of paid jobs since leaving full‐time education	2.4 (2.2)	2.8 (2.6)	−0.4 [−1.2,0.3]	*t*(154) = −1.2^NS^	−
If unemployed, duration of unemployment (months)	48.0 (26.4)	10.2 (15.2)	37.8 [12.3, 63.4]	*t*(14) = 3.2**	1.6
If unemployed, likelihood of gaining employment in next 12 months	2.1 (0.8)	4.3 (1.6)	−2.2 [−3.6, −0.9]	*t*(14) = −3.60**	−1.9
If employed, number of current jobs	1.1 (0.2)	1.2 (0.5)	−0.1 [−0.2,0.0]	*t*(129) = −1.5^NS^	−
If employed, duration of current employment (months)	38.7 (29.6)	29.8 (25.0)	8.9 [−0.5, 18.3]	*t*(129) = 1.9^NS^	−
If employed, number of hours worked per week	32.3 (14.7)	34.0 (12.5)	−1.6 [−6.4, 3.1]	*t*(129) = −0.7^NS^	−
If employed, whether in permanent employment (yes)	50 (83%)	60 (85%)	−	*χ* ^2^(1, *N* = 131) = 0.03, *p* = .855	−
If employed, part of private/company pension scheme	15 (25%)	24 (34%)	−	*χ* ^2^(1, *N* = 131) = 1.21, *p* = .272	
If in permanent employment, expect to still be in 12 months	36 (72%)	34 (57%)	−	*χ* ^2^(1, *N* = 110) = 2.77, *p* = .096	−

Note: NS, not significant, ^*^ *p* < .05, ^**^ *p* < .01.

### Experiences of gaining employment

Fewer young people with a history of DLD had used a CV to apply for jobs (DLD = 66 (83%); AMP = 82 (94%)), had attended an assessment centre as part of the interview process (DLD = 11 (14%); AMP = 31 (36%)), had attended a face‐to‐face interview (DLD = 69 (86%); AMP = 85 (98%)), or had given a telephone interview (DLD = 22 (28%); AMP = 39 (45%)), compared with their AMPs. Those young people with a history of DLD who had attended an assessment centre, face‐to‐face interview, or had a telephone interview, reported on a scale of 1–5, that they found all three situations more difficult when compared with the difficulty levels reported by AMPs who had attended an assessment centre. There was no difference between the groups on whether they had used the guaranteed interview scheme but 15% (*N* = 11) of young people with a history of DLD had asked for special arrangements at interview whilst this was not the case for any of the AMPs.

### Employment support and relationships

There was no difference between the groups on whether they had support from colleagues or managers, whether the support was formal, whether they had problems getting along with colleagues or managers, or their job satisfaction.

### Income

Table 9 presents data on annual income for adults with DLD and their peers. Ordered logistic regression was carried out to investigate the effect of occupational category and DLD status on income. This analysis was significant with a main effect of occupational category. Compared with elementary occupations, participants earned more in occupations in the categories: process, plant, and machine operatives (unstandardized *β* = 4.86 (95% CI = 2.56, 7.16), *p* < .001), skilled trades (unstandardized *β* = 1.99 (95% CI = 0.46, 3.52), *p* = .011), administrative and secretarial (unstandardized *β* = 1.98 (95% CI = 0.69, 3.28), *p* = .003), associate professional and technical (unstandardized *β* = 1.91 (95% CI = 0.34, 3.48), *p* = .017), professional (unstandardized *β* = 3.24 (95% CI = 1.72, 4.76), *p* < .001), managers, directors and senior officials (unstandardized *β* = 2.92 (95% CI = 0.97, 4.86), *p* = .002) but not sales and customer service (unstandardized *β* = 0.52 (95% CI = −0.66, 1.71, *p* = .388) or caring leisure and other service (unstandardized *β* = 0.93 (95% CI = −0.41, 2.28, *p* = .173). There was no main effect of DLD status, unstandardized *β* = 0.37 (95% CI = −0.29, 1.03), *p* = .275. These findings should be interpreted with the limited number of participants in some of the occupational categories in mind.

**Table 9:** Annual income

Income band	DLD, *n* (%)	AMP, *n* (%)
< £5200	9 (15%)	5 (7%)
£5201–£10,400	15 (25%)	13 (18%)
£10,401–£15,600	18 (30%)	20 (28%)
£15,601–£20,800	11 (18%)	14 (20%)
£20,801–£26,000	6 (10%)	12 (17%)
£26,001–£31,200	1 (1%)	7 (10%)

Note: There were 60 DLDs and 71 AMPs (131 in total) who provided data for income (currently in paid employment, full time or part time).

### Are there group differences in income based on education level achieved and/or type of employment?

We examined whether, at any employment level, there were group differences in level of income received (i.e., whether those with DLD were treated differently in this respect). Ordered logistic regressions showed that, whilst controlling for type of employment (full time, part time and other), income levels did not differ between groups, unstandardized *β* = 0.55 (95% CI = −0.09, 1.19), *p* = .095. Similarly, whilst controlling for highest educational qualification, income levels did not differ between groups, unstandardized *β* = 0.40 (95% CI = −0.31, 1.11), *p* = .270. In other words, for all measures available, level of pay did not vary according to group, i.e., income was the same for the same type of employment and for the same level of qualifications for individuals with and without a history of DLD.

### Concurrent relationships between profile of abilities and education/employment outcomes

The zero‐order correlations among highest qualification attained, employment type and abilities (language, reading, PIQ) are presented in table 10. For simplicity, education and vocational qualifications were amalgamated into one category: highest of either qualification.

**Table 10:** Correlation matrix between variables of interest

	1	2	3	4	5	6
1. Highest qualification	1					
2. Core language	Overall: 0.58*** DLD: 0.48*** AMP: 0.28*	1				
3. Overall reading	Overall: 0.59*** DLD: 0.44*** AMP: 0.43***	Overall: 0.85*** DLD: 0.80*** AMP: 0.60***	1			
4. PIQ	Overall: 0.50*** DLD: 0.43*** AMP: 0.29**	Overall: 0.60*** DLD: 0.52*** AMP: 0.32**	Overall: 0.58*** DLD: 0.45*** AMP: 0.41***	1		
5. Employment type	Overall: 0.45*** DLD: 0.14NS AMP: 0.47***	Overall: 0.34*** DLD: 0.14^NS^ AMP: 0.21^NS^	Overall: 0.37*** DLD: 0.13NS AMP: 0.40***	Overall: 0.23** DLD: 0.01NS AMP: 0.17NS	1	
6. GCSE score	Overall: 0.71*** DLD: 0.66*** AMP: 0.51***	Overall: 0.71*** DLD: 0.60*** AMP: 0.30*	Overall: 0.73*** DLD: 0.59*** AMP: 0.47***	Overall: 0.62*** DLD: 0.45*** AMP: 0.52***	Overall: 0.38*** DLD: 0.18NS AMP: 0.39**	1

Note: DLD, developmental language disorder; AMP, age‐matched peers; NS, not significant; ^*^ *p* < .05, ^**^ *p* < .01, ^***^ *p* < .001.

Higher educational/vocational qualifications were associated with better language, better reading and higher PIQ for the overall sample, as well as when correlations for the DLD and AMP groups were examined separately. For employment, correlations were generally weaker and significant mainly when the sample was examined as a whole. A more professional job was associated with higher qualifications, better language and reading and higher PIQ. However, it is important to note that none of the employment correlations were significant for the DLD group.

### Longitudinal relationships between GCSE performance and education/employment outcomes

Linear regression models were run to establish whether there was a relationship between performance at GCSE and PIQ (independent variables) and education and employment outcomes (dependent variables) at age 24. Group (DLD or AMP) was also included in the regression model as an independent variable to see if the effect observed differed by group. Thus, the predictors were GCSE score, PIQ, group, and the interaction between group and GCSE score.

For education (highest qualification obtained), the overall model was significant, *F*(4,140) = 37.53, *p* < .001, variance explained 50.4%, but the only significant predictor was GCSE score, unstandardized *β* = 0.01 [0.00, 0.01], *p* < .001, standardized *β* =  0.60. That is, those young people who scored higher GCSE grades had higher qualifications at age 24 and the effect remained after controlling for PIQ. The effect did not differ across groups.

For employment (job type) the overall model was significant, *F*(4,102) = 5.91, *p* < .001, variance explained 15.6%. There were no significant individual predictors.

## Discussion

The educational and employment status of individuals as they enter early adulthood are real‐world measures of how adequately they are prepared for the rest of their lives. Many factors bear on these developmental outcomes, including the individual's abilities, the personal support he or she has received, the educational and training opportunities that he or she has experienced, and the equitability of the occupational structures available. In this investigation, we sought to ascertain how a large sample of young people with histories of DLD fare in these regards. The findings afford rich evidence on early adulthood outcomes in people with this hidden disability.

It should be recognized first that, at the group level, those with histories of DLD remain disadvantaged in respect of linguistic and non‐linguistic abilities as they enter adulthood. There were significant differences between the DLD group and their AMPs without DLD on standard measures of receptive and expressive language, reading ability, and nonverbal IQ. At the individual level, consistent with abundant evidence on the heterogeneity of DLD (Conti‐Ramsden 2008, Conti‐Ramsden and Durkin 2016), there was considerable variation, with some participants continuing to lag their peers markedly but others now scoring within the normal range; these individual differences, we will discuss below, certainly bear on educational and employment outcomes. We also reiterate that in order to make meaningful comparisons between participants with DLD and their AMP we put in place a targeted participant strategy ensuring that individuals in the AMP group came from a broad background which afforded comparability but also yielded variation including a broad spread of language abilities in the AMP comparison sample.

### Educational outcomes

In terms of academic qualifications, participants with DLD were less likely than AMPs to have achieved higher level outcomes, such as A‐Levels (18% versus 72%, respectively) or university degrees (10% versus 41%). In terms of vocational qualifications, participants with DLD were less likely to have achieved outcomes at Level 4 or above (1% versus 10%). Thus, at the group level, outcomes are clearly less favourable to those with DLD. Yet, there are exceptions, with smaller but not trivial proportions of participants reaching these levels successfully. It should be acknowledged that we do not have more specific grade information (i.e., A‐Level passes are scored on a six‐point scale, and degree classes in the UK also range across four tiers) and that educational outcomes were not independently validated with direct data. This is a limitation of this study that calls for future research to include verification of reported data on educational qualifications obtained. Few participants pursued vocational qualifications above Level 3. There were more participants with DLD who had achieved lower levels of vocational qualification.

Although comparisons with peers without DLD continue to reveal disadvantages to those with DLD at the group level, it is important to underline the improvement in educational outcomes that appear to be occurring in the 21st century. Recall that studies involving young adults with DLD in the 1990s revealed most young people had left compulsory education with no qualifications and they had continued to remain outside education into their adulthood (Clegg *et al*. 2005, Mawhood *et al*. 2000, Records *et al*. 1992). Opportunities for vocational education have also increased since the latter part of the last millennium (Eichhorst *et al*. 2015). Nearly half (45%) of the young people with a history of DLD in our MLS sample had successfully achieved at least a Level 3 academic or vocational qualification by age 24. Such qualifications place the individual eligible for advanced study or training.

In respect of current educational status, a lower proportion (19%) of the DLD group than in the AMP group (33%) reported being in education during the last 6 months. This indicates an advantage to the AMPs, but it is notable that a sizeable minority of each group was still studying. Thus, again, there is continuing heterogeneity of educational progress in DLD.

We found a strong relationship between GCSE score (obtained at around age 16) and educational outcomes (highest qualification obtained) at age 24, with the former explaining 50% of the variance in the latter. GCSE performance was strongly correlated with contemporaneous language and literacy scores, even after controlling for PIQ—but this relationship held for both groups. Language ability is, unsurprisingly, a strong influence on educational progress at school and, as shown in this study, on educational qualifications obtained at college or University post compulsory secondary education. The pattern of achievement established by around the GCSE stage is a good predictor of subsequent achievement.

Some young adults with a history of DLD achieved very well academically. They obtained University undergraduate degrees and some entered postgraduate study. A total of 10% of young adults with DLD obtained this level of qualifications and a further 18% continued to engage in education and were studying at this age. This is broadly in line with figures reported in a different educational system in Canada, where 27% of participants with DLD completed a university degree (Johnson *et al*. 2010).

### Employment outcomes

The employment histories of the participants showed some similarities between groups and some telling differences. There were similar patterns in respect of number of paid jobs since leaving full‐time education, the number of current jobs, the duration of current employment, the number of hours per week in the main job, whether employment was permanent (with over 80% of both groups reporting permanent status), and whether or not the individual was enlisted in a pension scheme. The NEET figures are also less disturbing than reported in earlier generations. A small proportion of individuals in both groups were NEET. The proportion of young adults (approximately 12%) with DLD who were NEET was similar to that of AMPs and in line with figures for the UK generally (9%; Department for Education 2015). Similar recent findings have been reported by other researchers in the UK (Carroll and Dockrell 2010).

There was, however, a difference in the proportions of each group represented in professional roles and in less skilled employment, with the group with DLD represented less frequently in the former and more frequently in the latter. This calls for attention. As noted above, the two groups, by definition and as confirmed in contemporaneous assessments, were not equivalent in linguistic/ literacy abilities. This may well influence their choices of employment and their likelihood of being selected. It is too early to tell whether this foreshadows a continuing or exacerbating differential in employment progress. There is independent evidence that shifts may take place further into the 20s. Beitchman *et al*. (2001) and Johnson *et al*. (2010), in their Canadian longitudinal sample of individuals with DLD, found that at age 19 there were similarities in terms of the proportion of young people in employment but the group with DLD showed poorer occupational outcomes than their peers by age 25. Our participants were only slightly younger than this. Future research is needed to examine career trajectories beyond early adulthood.

It is notable that—in contrast to the regression analysis findings in respect of educational outcomes at age 24, where GCSE score accounted for half of the variance—for employment (job type) the amount of variance explained by GCSE and PIQ was modest and no individual predictor could be identified. Our interpretation is that macro‐level changes in the provision of educational opportunities and the regulation of workplace entitlements are beginning to show some positive effects for young people with DLD. At age 19 years, the main activity of these same young people from the MLS was education and training (Durkin *et al*. 2009); Very encouragingly, now in young adulthood, these individuals have secured employment on a scale approaching that of their peers without DLD (66% of those with DLD employed and 73% of AMPs). The most frequent main activity of young adults with or without DLD is employment. The proportion of individuals at work is in line with national statistics for the proportion of people aged from 16 to 64 in employment in the UK in 2015, which was calculated at 73% (ONS 2015).

### Income levels

Little previous evidence was available on the relative income levels of young adults with DLD, compared with AMPs, and what is available was carried out in the latter part of the past millennium when employment environment was different than in the 2010s. Records *et al*. (1992), in a smaller sample including a higher proportion of college students, found no evidence of income differences). The present findings, based on a larger sample, the majority of whom were no longer students, found no significant differences across groups when examining six income bands. When these were amalgamated into low, middle and high income, the findings indicated an association between high income and group.

However, when we examined income level as a function of group for each level of employment status (full time, part time, professional) or for each level of academic or vocational qualification, we found no differences. That is, there was no evidence that within a particular category of employment participants with DLD were suffering a disadvantageous pay differential. An optimistic interpretation of the finding that income levels did not differ significantly between groups is that the recent Equality Act 2010 is regulating effectively how young workers with disabilities are compensated.

On the other hand, the participants are in early stages of their working lives and most are still at low salary levels, so divergence of rewards may not yet have emerged. To test the adequacy of legislative controls more fully would require larger samples with information regarding more specific job titles and job specifications; to test the durability of what appears at present to be reasonably equitable pay would require longitudinal studies extending further into adult lives. What can be concluded for the moment is that many young people with DLD are entering the world of work on comparable income terms to their peers without DLD.

### Employment search

The participants’ reports on their experiences in the course of job applications and in relation to employment support are revealing. Although a clear majority of both groups did use a CV to apply for jobs, some 17% of the DLD group did not do so (versus 6% of the AMPs). In some cases, this may reflect lack of preparation/ application skills; in others, it may mean that the jobs being applied for were such that formal CVs were not required by the employers. It is important to note, however, that although adults with DLD had significantly more institutional support (e.g., help with writing), this support did not seem to translate into preparation and use of a CV when seeking employment for approximately one‐sixth of applicants with DLD. The relative figures were similar for attending face‐to‐face interviews (86% DLD, 98% AMPs); that is, most people did undertake this stage but, of those who did not do so, most were participants with DLD. Participants with DLD were much less likely (28%) to have had a telephone interview than were AMPs (45%). Only 14% of the DLD group attended assessment centres, while 36% of AMPs did. The DLD participants found the assessment centre's processes, face‐to‐face interviews, and telephone interviews significantly more difficult than did the AMPs.

Interestingly, only a small proportion (15%) of participants with DLD had requested special arrangements at interviews and only three individuals had taken advantage of the guaranteed interview scheme. These findings along with the figures in relation to the use of a CV to apply for jobs suggests there is room for more focused preparation as well as raising awareness among young people with DLD regarding strategies, options and rights regarding job applications and interviewing. It is open to speculation whether job seekers were aware of their options or entitlements in these regards, or whether they were reluctant to draw attention to characteristics that they felt might affect their prospects. Overall, the indications are that greater proportions of those with DLD than those without DLD are less well prepared for the competitive environment of job seeking.

## Conclusions

An important message of this study is that, notwithstanding the difficulties that they face, there are positive aspects to early adult outcomes for young people with DLD who were identified relatively early in their lives and had access to arguably the best services the UK had to offer at that time (all individuals with DLD attended language units). The wide range of educational qualifications obtained demonstrates that the heterogeneity of outcomes of individuals with DLD observed in adolescence (Conti‐Ramsden *et al*. 2009, Dockrell *et al*. 2007, Durkin *et al*. 2009) continues in young adulthood.

Challenges remain. History of employment data revealed that young adults with DLD who were unemployed had been so considerably longer than their peers (on average four times longer) and they were less optimistic about securing employment the following year. It appears that if individuals with DLD do not secure employment by young adulthood or have experienced years of unemployment, they are likely to feel their prospects for future employment are poor. It is also of note that a higher proportion of AMPs was in full‐time employment. Recent research has suggested that improved educational opportunities followed by lack of full‐time employment might associate with mental health difficulties in this DLD group (Botting *et al*. 2016, Conti‐Ramsden *et al*. 2016).

In sum, in this longitudinal, UK‐based study, the overall picture of educational and early employment outcomes for young adults with DLD indicates ‘disadvantage but not disaster.’ At the group level, there are certainly less favourable outcomes for those with DLD. But, compared with evidence from earlier studies with very poor educational outcomes and high levels of unemployment, there are indicators of improvements and there are individual differences; most notably, all participants with DLD achieved some qualifications, many obtained some form of employment, NEET rates were no higher than for the population in general, and a minority entered professional careers. This suggests that some progress has been made in terms of how young people with DLD are prepared for crucial aspects of entry into adulthood.

## Notes

1 See https://www.gov.uk/government/news/lowest-rate-of-young-people-neet-for-20-years/.

2 A new grading system will be introduced whereby letter grades are replaced by numbers 1‐9 (see https://www.gov.uk/government/uploads/system/uploads/attachment_data/file/537147/Postcard_-_Grading_New_GCSEs.pdf).

3 See http://www.legislation.gov.uk/ukpga/2010/15/contents/.

4 See http://www.accreditedqualifications.org.uk/qualifications-and-credit-framework-qcf.html/.

5 See http://www.jobs.ac.uk/careers-advice/interview-tips/1821/surviving-the-assessment-centre.
